# 1-[(4-Chloro­phen­yl)(phenyl­imino)­meth­yl]-7-meth­oxy-2-naphthol–1,4-diaza­bicyclo­[2.2.2]octane (2/1)

**DOI:** 10.1107/S1600536810034690

**Published:** 2010-09-04

**Authors:** Atsushi Nagasawa, Ryosuke Mitsui, Yuichi Kato, Akiko Okamoto, Noriyuki Yonezawa

**Affiliations:** a Department of Organic and Polymer Materials Chemistry, Tokyo University of Agriculture & Technology, 2-24-16 Naka-machi, Koganei, Tokyo 184-8588, Japan

## Abstract

In the crystal structure of the title cocrystal, 2C_24_H_18_ClNO_2_·C_6_H_12_N_2_, the 1,4-diaza­bicyclo­[2.2.2]octane mol­ecule is located on a twofold rotation axis and linked to the two triaryl­imine mol­ecules by O—H⋯N hydrogen bonds, forming a 2:1 aggregate. C—H⋯Cl inter­actions are also observed. In the triaryl­imine mol­ecule, the naphthalene ring system makes dihedral angles of 80.39 (6) and 82.35 (6)°, respectively, with the phenyl and benzene rings. The dihedral angle between these two latter rings is 87.09 (7)°.

## Related literature

For our study of the electrophilic aromatic aroylation of 2,7-dimethoxynaphthalene and *peri-*aroyl­naphthalene compounds, see: Okamoto & Yonezawa (2009 ▶). For related structures, see: Hijikata *et al.* (2010 ▶); Mitsui, Nakaema, Noguchi & Yonezawa (2008 ▶); Mitsui, Nakaema, Noguchi, Okamoto & Yonezawa (2008 ▶); Watanabe, Nakaema, Muto *et al.* (2010 ▶); Watanabe, Nakaema, Nishijima *et al.* (2010 ▶). 
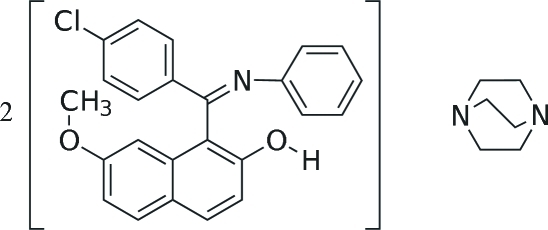


## Experimental

### 

#### Crystal data

C_24_H_18_ClNO_2_·0.5C_6_H_12_N_2_
*M* *_r_* = 443.93
Monoclinic, 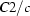

*a* = 25.0027 (5) Å
*b* = 9.92298 (18) Å
*c* = 20.0052 (4) Å
β = 114.621 (1)°
*V* = 4512.07 (16) Å^3^
*Z* = 8
Cu *K*α radiation
μ = 1.71 mm^−1^
*T* = 193 K
0.60 × 0.50 × 0.40 mm

#### Data collection

Rigaku R-AXIS RAPID diffractometer
Absorption correction: numerical (*NUMABS*; Higashi, 1999 ▶) *T*_min_ = 0.381, *T*_max_ = 0.548
39753 measured reflections
4125 independent reflections
3831 reflections with *I* > 2σ(*I*)
*R*_int_ = 0.026

#### Refinement

*R*[*F* ^2^ > 2σ(*F* ^2^)] = 0.034
*wR*(*F* ^2^) = 0.097
*S* = 1.04
4125 reflections
295 parameters
H atoms treated by a mixture of independent and constrained refinement
Δρ_max_ = 0.44 e Å^−3^
Δρ_min_ = −0.32 e Å^−3^

### 

Data collection: *PROCESS-AUTO* (Rigaku, 1998 ▶); cell refinement: *PROCESS-AUTO*; data reduction: *CrystalStructure* (Rigaku/MSC, 2004 ▶); program(s) used to solve structure: *SIR2004* (Burla *et al.*, 2005 ▶); program(s) used to refine structure: *SHELXL97* (Sheldrick, 2008 ▶); molecular graphics: *ORTEPIII* (Burnett & Johnson, 1996 ▶); software used to prepare material for publication: *SHELXL97*.

## Supplementary Material

Crystal structure: contains datablocks I, global. DOI: 10.1107/S1600536810034690/is2593sup1.cif

Structure factors: contains datablocks I. DOI: 10.1107/S1600536810034690/is2593Isup2.hkl

Additional supplementary materials:  crystallographic information; 3D view; checkCIF report

## Figures and Tables

**Table 1:** Hydrogen-bond geometry (Å, °)

*D*—H⋯*A*	*D*—H	H⋯*A*	*D*⋯*A*	*D*—H⋯*A*
O1—H1⋯N2^i^	0.89 (2)	1.86 (2)	2.7401 (18)	167.2 (18)
C20—H20⋯Cl1^ii^	0.95	2.78	3.6071 (17)	146

Symmetry codes: (i) 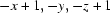; (ii) 
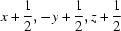
.

**Fig. 1.**
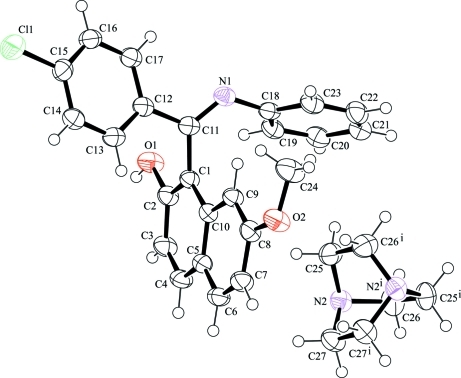
The asymmetric unit of the cocrystal of triarylimine and DABCO, showing 50% probability displacement ellipsoids [symmetry code: (i) 1 - x, y, 3/2 - z].

**Fig. 2.**
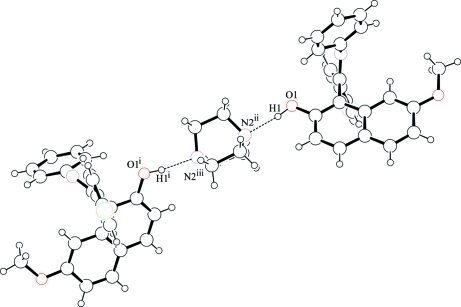
The 2:1 comolecular unit of triarylimines and DABCO [symmetry codes: (i) 1 - x, y, -z; (ii) 1 - x, -y, 1 - z; (iii) x, -y, -1/2 + z]. The intermolecular O—H···N hydrogen bond are shown as dashed lines.

**Fig. 3.**
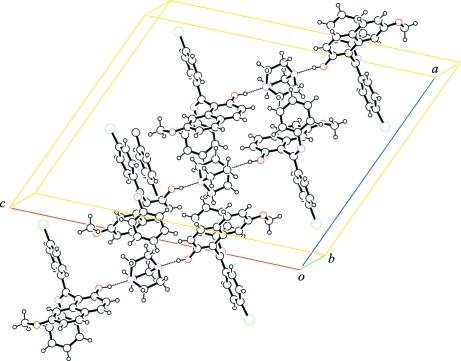
A partial crystal packing diagram of the cocrystal of triarylimine and DABCO, viewed down the b axis. The intermolecular O—H···N hydrogen bond are shown as dashed lines.

## supplementary crystallographic information

### Comment

In the course of our study on electrophilic aromatic aroylation of 2,7-dimethoxynaphthalene, *peri-*aroylnaphthalene compounds have proven to be formed regioselectively with the aid of suitable acidic mediators (Okamoto & Yonezawa, 2009). Recently, we reported the crystal structures of several 1,8-diaroylated naphthalene homologues exemplified by bis(4-bromobenzoyl)(2,7-dimethoxynaphthalene-1,8-diyl)dimethanone (Watanabe, Nakaema, Muto *et al.*, 2010).

The aroyl groups in these compounds are bonded almost perpendicularly to the naphthalene rings at the 1,8-positions but the benzene ring moieties of the aroyl groups tilt slightly toward the *exo* sides of the naphthalene rings. Moreover, the X-ray crystal structural analyses of 1-(4-substituted benzoylated)naphthalenes, *i.e.*, 1-(4-chlorobenzoyl)-2,7-dimethoxynaphthalene (Mitsui, Nakaema, Okamoto & Yonezawa, 2008), 1-(4-nitrobenzoyl)-2,7-dimethoxynaphthalene (Watanabe, Nakaema, Nishijima *et al.*, 2010) and methyl 4-(2,7-dimethoxy-1-naphthoyl)benzoate (Hijikata *et al.*, 2010), have also revealed essentially the same non-coplanar structure as the 1,8-diaroylated naphthalenes. Contrarily, the benzene ring of (4-chlorophenyl)(2-hydroxy-7-methoxynaphthalen-1-yl)methanone (Mitsui, Nakaema, Noguchi & Yonezawa, 2008) is bonded to the naphthalene ring with nearly coplanar configuration in the opposite direction against the 2-hydroxy group. This crystal structure is stabilized by intramolecular hydrogen bond between 2-hydroxy group and the carbonyl group.

As a part of our continuous study on the molecular structures of this kind of homologous molecules, we have investigated imination of aroylated naphthalene derivatives. Triarylimine has been clarified to be synthesized effectively by imination with the aid of TiCl_4_ and 1,4-diazabicyclo[2.2.2]octane (DABCO). The cocrystal of triarylimines and DABCO (2/1) was prepared directly from the reaction mixture.

An *ORTEPIII* (Burnett & Johnson, 1996) plot of the title cocrystal is shown in Fig. 1. In the crystal packing, one DABCO molecule is connected to two triarylimine molecules with two O—H···N hydrogen bonds (Fig. 2). The 2:1 comolecular unit of triarylimines and DABCO have twofold rotation symmetry, with the central DABCO molecule lying on the rotation axis. In triarylimine of the comolecular unit, the interplanar angles of phenyl ring (C18–C23) attached to nitrogen atom (N1) and benzene ring (C12–C17) attached to carbon atom (C11) against the naphthalene ring (C1–C10) are 80.39 (6) and 82.35 (6)°, respectively. Furthermore, the interplanar angle between the phenyl and benzene rings is 87.09 (7)°.

The molecular packing is mainly stabilized by intermolecular hydrogen bonds and van der Waals interactions. Triarylimine and DABCO are linked with intermolecular O—H···N hydrogen bond [O1—H1···N2 = 1.86 (2) Å]. The 2:1 comolecular units are aligned along the *a* axis (Fig. 3). The C—H···π interaction between the methylene hydrogen atom of DABCO molecule and the naphthalene ring is observed along the *c* axis [C5···H25A = 2.69 Å]. Furthermore, the hydrogen bond between the chlorine atom and the hydrogen atom of the phenyl ring (C18–C23) is observed along the *b* axis [Cl1···H20 = 2.78 Å].

### Experimental

To a solution of 1-(4-chlorobenzoyl)-2-hydroxy-7-methoxynaphthalene (0.2 mmol, 62.8 mg) in chlorobenzene (1 ml), a mixture of aniline (0.22 mmol, 20.5 mg), TiCl_4_ (0.33 mmol, 62.4 mg), DABCO (1.32 mmol, 148.0 mg) and chlorobenzene (1 ml) was added by portions at 363 K under nitrogen atmosphere. After the reaction mixture was stirred at 398 K for 1.5 h, the resulting solution was filtrated to remove the precipitate. The solvent was removed under reduced pressure to give crude material. The crude material thus obtained was subjected to crystallization from CHCl_3_/*n*-hexane to give the cocrystal of triarylimines and DABCO (2/1) as colorless block (m.p. 445.6–446.0 K, yield 19.5 mg, 22%).

Spectroscopic Data: ^1^H NMR (300 MHz, DMSO-*d_6_*) δ; 10.13, (s, 1H), 7.66–7.60 (m, 4H), 7.44 (d, 2H), 7.00 (t, 2H), 6.95 (d, 1H), 6.86–6.76 (m, 4H), 6.52 (d, 1H), 3.64 (s, 3H), 3.29 (s, 6H); ^13^C NMR (75 MHz, DMSO-*d_6_*) 164.4, 158.2, 153.7, 151.0, 137.6, 135.7, 132.2, 130.3, 130.0, 129.7, 128.7, 128.2, 123.8, 122.9, 119.2, 115.1, 115.0, 114.9, 102.6, 55.1, 47.3; IR (KBr): 3407, 2937, 2592, 1625, 1585, 1509, 1227; HRMS (*m/z*): [*M* + H]^+^ calcd for C_24_H_19_ClNO_2_, 388.1110; found, 388.1104.

### Refinement

All the H-atoms could be located in difference Fourier maps. The O—H hydrogen atom was freely refined: O1—H1 = 0.89 (2) Å. The C-bound H-atoms were subsequently refined as riding atoms, with C—H = 0.95 (aromatic) and 0.98 (methyl) Å, and *U*_iso_(H) = 1.2*U*_eq_(C).

### Crystal data

**Table:**

C_24_H_18_ClNO_2_·0.5C_6_H_12_N_2_	*F*(000) = 1864
*M**_r_* = 443.93	*D*_x_ = 1.307 Mg m^−^^3^
Monoclinic, *C*2/*c*	Melting point = 445.6–446.0 K
Hall symbol: -C 2yc	Cu *K*α radiation, λ = 1.54187 Å
*a* = 25.0027 (5) Å	Cell parameters from 33487 reflections
*b* = 9.92298 (18) Å	θ = 3.6–68.2°
*c* = 20.0052 (4) Å	µ = 1.71 mm^−^^1^
β = 114.621 (1)°	*T* = 193 K
*V* = 4512.07 (16) Å^3^	Block, colorless
*Z* = 8	0.60 × 0.50 × 0.40 mm

### Data collection

**Table:**

Rigaku R-AXIS RAPID diffractometer	4125 independent reflections
Radiation source: rotating anode	3831 reflections with *I* > 2σ(*I*)
graphite	*R*_int_ = 0.026
Detector resolution: 10.00 pixels mm^-1^	θ_max_ = 68.2°, θ_min_ = 3.9°
ω scans	*h* = −30→30
Absorption correction: numerical (*NUMABS*; Higashi, 1999)	*k* = −11→11
*T*_min_ = 0.381, *T*_max_ = 0.548	*l* = −24→24
39753 measured reflections	

### Refinement

**Table:**

Refinement on *F*^2^	Secondary atom site location: difference Fourier map
Least-squares matrix: full	Hydrogen site location: inferred from neighbouring sites
*R*[*F*^2^ > 2σ(*F*^2^)] = 0.034	H atoms treated by a mixture of independent and constrained refinement
*wR*(*F*^2^) = 0.097	*w* = 1/[σ^2^(*F*_o_^2^) + (0.0539*P*)^2^ + 2.6288*P*] where *P* = (*F*_o_^2^ + 2*F*_c_^2^)/3
*S* = 1.04	(Δ/σ)_max_ < 0.001
4125 reflections	Δρ_max_ = 0.44 e Å^−^^3^
295 parameters	Δρ_min_ = −0.32 e Å^−^^3^
0 restraints	Extinction correction: *SHELXL97* (Sheldrick, 2008), Fc^*^=kFc[1+0.001xFc^2^λ^3^/sin(2θ)]^-1/4^
Primary atom site location: structure-invariant direct methods	Extinction coefficient: 0.00076 (6)

### Special details

**Table:**

Geometry. All e.s.d.'s (except the e.s.d. in the dihedral angle between two l.s. planes) are estimated using the full covariance matrix. The cell e.s.d.'s are taken into account individually in the estimation of e.s.d.'s in distances, angles and torsion angles; correlations between e.s.d.'s in cell parameters are only used when they are defined by crystal symmetry. An approximate (isotropic) treatment of cell e.s.d.'s is used for estimating e.s.d.'s involving l.s. planes.
Refinement. Refinement of *F*^2^ against ALL reflections. The weighted *R*-factor *wR* and goodness of fit *S* are based on *F*^2^, conventional *R*-factors *R* are based on *F*, with *F* set to zero for negative *F*^2^. The threshold expression of *F*^2^ > σ(*F*^2^) is used only for calculating *R*-factors(gt) *etc*. and is not relevant to the choice of reflections for refinement. *R*-factors based on *F*^2^ are statistically about twice as large as those based on *F*, and *R*- factors based on ALL data will be even larger.

### Fractional atomic coordinates and isotropic or equivalent isotropic displacement parameters (Å^2^)

**Table:**

	*x*	*y*	*z*	*U*_iso_*/*U*_eq_	
Cl1	0.091242 (19)	−0.01553 (5)	0.13553 (2)	0.06399 (16)	
O1	0.39706 (5)	0.09386 (10)	0.35831 (6)	0.0414 (3)	
O2	0.27685 (5)	−0.16378 (10)	0.60663 (6)	0.0454 (3)	
N1	0.30621 (5)	0.22752 (11)	0.43787 (6)	0.0334 (3)	
N2	0.52801 (5)	−0.05890 (12)	0.70856 (6)	0.0364 (3)	
C1	0.35467 (5)	0.01095 (12)	0.43518 (7)	0.0285 (3)	
C2	0.39665 (6)	−0.00014 (13)	0.40731 (7)	0.0320 (3)	
C3	0.43819 (6)	−0.10588 (14)	0.43064 (8)	0.0372 (3)	
H3	0.4684	−0.1102	0.4136	0.045*	
C4	0.43495 (6)	−0.20190 (14)	0.47761 (8)	0.0369 (3)	
H4	0.4623	−0.2743	0.4916	0.044*	
C5	0.39191 (6)	−0.19608 (13)	0.50592 (7)	0.0319 (3)	
C6	0.38745 (6)	−0.29572 (14)	0.55401 (8)	0.0381 (3)	
H6	0.4123	−0.3726	0.5650	0.046*	
C7	0.34832 (7)	−0.28366 (14)	0.58474 (8)	0.0393 (3)	
H7	0.3458	−0.3518	0.6167	0.047*	
C8	0.31135 (6)	−0.16910 (14)	0.56896 (7)	0.0345 (3)	
C9	0.31213 (6)	−0.07365 (13)	0.51986 (7)	0.0311 (3)	
H9	0.2857	0.0004	0.5079	0.037*	
C10	0.35246 (5)	−0.08518 (12)	0.48682 (7)	0.0283 (3)	
C11	0.30795 (5)	0.11744 (13)	0.40603 (7)	0.0296 (3)	
C12	0.25612 (5)	0.08458 (13)	0.33587 (7)	0.0306 (3)	
C13	0.23887 (6)	−0.04876 (14)	0.31739 (7)	0.0347 (3)	
H13	0.2624	−0.1194	0.3474	0.042*	
C14	0.18793 (6)	−0.08022 (14)	0.25584 (8)	0.0370 (3)	
H14	0.1758	−0.1712	0.2442	0.044*	
C15	0.15531 (6)	0.02355 (15)	0.21194 (7)	0.0378 (3)	
C16	0.17224 (6)	0.15721 (15)	0.22735 (8)	0.0396 (3)	
H16	0.1498	0.2271	0.1955	0.048*	
C17	0.22236 (6)	0.18705 (14)	0.28987 (7)	0.0350 (3)	
H17	0.2339	0.2783	0.3016	0.042*	
C18	0.35302 (6)	0.26745 (13)	0.50447 (7)	0.0321 (3)	
C19	0.41007 (6)	0.28720 (13)	0.51060 (8)	0.0355 (3)	
H19	0.4198	0.2637	0.4710	0.043*	
C20	0.45241 (6)	0.34079 (14)	0.57415 (8)	0.0396 (3)	
H20	0.4910	0.3558	0.5775	0.048*	
C21	0.43962 (7)	0.37310 (15)	0.63327 (8)	0.0414 (3)	
H21	0.4692	0.4094	0.6770	0.050*	
C22	0.38341 (7)	0.35180 (15)	0.62785 (8)	0.0423 (3)	
H22	0.3744	0.3725	0.6684	0.051*	
C23	0.34011 (6)	0.30065 (14)	0.56397 (8)	0.0385 (3)	
H23	0.3014	0.2880	0.5605	0.046*	
C24	0.24275 (8)	−0.04438 (17)	0.59824 (10)	0.0523 (4)	
H24A	0.2229	−0.0483	0.6313	0.063*	
H24B	0.2133	−0.0374	0.5473	0.063*	
H24C	0.2686	0.0345	0.6103	0.063*	
C25	0.47037 (7)	0.00787 (16)	0.67146 (8)	0.0437 (3)	
H25A	0.4462	−0.0418	0.6259	0.052*	
H25B	0.4761	0.1008	0.6576	0.052*	
C26	0.56208 (7)	0.01255 (17)	0.77789 (9)	0.0462 (4)	
H26A	0.5679	0.1075	0.7673	0.055*	
H26B	0.6013	−0.0299	0.8030	0.055*	
C27	0.51789 (7)	−0.19821 (15)	0.72649 (9)	0.0432 (3)	
H27A	0.5562	−0.2434	0.7538	0.052*	
H27B	0.4962	−0.2491	0.6805	0.052*	
H1	0.4247 (9)	0.075 (2)	0.3425 (11)	0.069 (6)*	

### Atomic displacement parameters (Å^2^)

**Table:**

	*U*^11^	*U*^22^	*U*^33^	*U*^12^	*U*^13^	*U*^23^
Cl1	0.0550 (3)	0.0596 (3)	0.0526 (3)	0.00847 (19)	−0.0022 (2)	−0.00729 (19)
O1	0.0492 (6)	0.0401 (5)	0.0517 (6)	0.0113 (4)	0.0378 (5)	0.0121 (4)
O2	0.0642 (7)	0.0358 (5)	0.0564 (6)	0.0060 (5)	0.0453 (6)	0.0087 (5)
N1	0.0348 (6)	0.0318 (6)	0.0381 (6)	0.0051 (4)	0.0197 (5)	0.0033 (5)
N2	0.0388 (6)	0.0394 (6)	0.0383 (6)	0.0020 (5)	0.0233 (5)	0.0011 (5)
C1	0.0299 (6)	0.0273 (6)	0.0310 (6)	0.0027 (5)	0.0154 (5)	0.0006 (5)
C2	0.0356 (7)	0.0306 (7)	0.0353 (7)	0.0021 (5)	0.0202 (6)	0.0017 (5)
C3	0.0366 (7)	0.0392 (8)	0.0454 (7)	0.0071 (6)	0.0265 (6)	0.0017 (6)
C4	0.0375 (7)	0.0348 (7)	0.0418 (7)	0.0120 (6)	0.0199 (6)	0.0028 (6)
C5	0.0357 (6)	0.0296 (7)	0.0317 (6)	0.0040 (5)	0.0151 (5)	0.0002 (5)
C6	0.0472 (8)	0.0288 (7)	0.0405 (7)	0.0091 (6)	0.0204 (6)	0.0050 (5)
C7	0.0549 (8)	0.0289 (7)	0.0404 (7)	0.0031 (6)	0.0261 (7)	0.0069 (6)
C8	0.0424 (7)	0.0313 (7)	0.0370 (7)	−0.0009 (5)	0.0236 (6)	0.0002 (5)
C9	0.0341 (6)	0.0284 (6)	0.0351 (6)	0.0029 (5)	0.0187 (5)	0.0014 (5)
C10	0.0295 (6)	0.0273 (6)	0.0294 (6)	0.0003 (5)	0.0135 (5)	−0.0013 (5)
C11	0.0328 (6)	0.0298 (6)	0.0348 (6)	0.0039 (5)	0.0224 (5)	0.0066 (5)
C12	0.0338 (6)	0.0321 (7)	0.0343 (6)	0.0042 (5)	0.0225 (5)	0.0038 (5)
C13	0.0362 (7)	0.0322 (7)	0.0384 (7)	0.0052 (5)	0.0183 (6)	0.0059 (5)
C14	0.0384 (7)	0.0346 (7)	0.0414 (7)	0.0016 (6)	0.0202 (6)	−0.0007 (6)
C15	0.0362 (7)	0.0478 (8)	0.0324 (7)	0.0070 (6)	0.0173 (6)	−0.0004 (6)
C16	0.0454 (8)	0.0399 (8)	0.0365 (7)	0.0133 (6)	0.0200 (6)	0.0082 (6)
C17	0.0424 (7)	0.0316 (7)	0.0370 (7)	0.0052 (5)	0.0225 (6)	0.0043 (5)
C18	0.0363 (7)	0.0267 (6)	0.0374 (7)	0.0074 (5)	0.0193 (5)	0.0051 (5)
C19	0.0397 (7)	0.0320 (7)	0.0430 (7)	0.0040 (5)	0.0256 (6)	0.0001 (5)
C20	0.0358 (7)	0.0344 (7)	0.0519 (8)	0.0027 (6)	0.0215 (6)	−0.0014 (6)
C21	0.0455 (8)	0.0357 (7)	0.0412 (7)	0.0047 (6)	0.0160 (6)	−0.0004 (6)
C22	0.0547 (9)	0.0394 (8)	0.0413 (7)	0.0054 (6)	0.0286 (7)	0.0003 (6)
C23	0.0393 (7)	0.0385 (8)	0.0463 (8)	0.0057 (6)	0.0263 (6)	0.0024 (6)
C24	0.0678 (10)	0.0459 (9)	0.0680 (10)	0.0119 (8)	0.0528 (9)	0.0094 (8)
C25	0.0489 (8)	0.0494 (9)	0.0387 (7)	0.0110 (7)	0.0240 (7)	0.0070 (6)
C26	0.0420 (8)	0.0565 (10)	0.0474 (8)	−0.0107 (7)	0.0259 (7)	−0.0084 (7)
C27	0.0516 (8)	0.0380 (8)	0.0494 (8)	0.0043 (6)	0.0305 (7)	0.0005 (6)

### Geometric parameters (Å, °)

**Table:**

Cl1—C15	1.7357 (15)	C13—H13	0.9500
O1—C2	1.3563 (16)	C14—C15	1.378 (2)
O1—H1	0.89 (2)	C14—H14	0.9500
O2—C8	1.3623 (15)	C15—C16	1.387 (2)
O2—C24	1.4282 (18)	C16—C17	1.384 (2)
N1—C11	1.2744 (17)	C16—H16	0.9500
N1—C18	1.4150 (17)	C17—H17	0.9500
N2—C26	1.4723 (19)	C18—C19	1.3937 (19)
N2—C25	1.4749 (18)	C18—C23	1.3964 (18)
N2—C27	1.4762 (19)	C19—C20	1.378 (2)
C1—C2	1.3828 (17)	C19—H19	0.9500
C1—C10	1.4241 (17)	C20—C21	1.385 (2)
C1—C11	1.5012 (17)	C20—H20	0.9500
C2—C3	1.4119 (19)	C21—C22	1.380 (2)
C3—C4	1.364 (2)	C21—H21	0.9500
C3—H3	0.9500	C22—C23	1.382 (2)
C4—C5	1.4106 (18)	C22—H22	0.9500
C4—H4	0.9500	C23—H23	0.9500
C5—C6	1.4159 (19)	C24—H24A	0.9800
C5—C10	1.4198 (17)	C24—H24B	0.9800
C6—C7	1.360 (2)	C24—H24C	0.9800
C6—H6	0.9500	C25—C26^i^	1.540 (2)
C7—C8	1.4154 (19)	C25—H25A	0.9900
C7—H7	0.9500	C25—H25B	0.9900
C8—C9	1.3706 (18)	C26—C25^i^	1.540 (2)
C9—C10	1.4221 (17)	C26—H26A	0.9900
C9—H9	0.9500	C26—H26B	0.9900
C11—C12	1.4966 (18)	C27—C27^i^	1.545 (3)
C12—C13	1.3931 (19)	C27—H27A	0.9900
C12—C17	1.3949 (18)	C27—H27B	0.9900
C13—C14	1.3890 (19)		
			
C2—O1—H1	110.6 (13)	C16—C15—Cl1	119.57 (11)
C8—O2—C24	116.85 (10)	C17—C16—C15	118.87 (13)
C11—N1—C18	121.58 (11)	C17—C16—H16	120.6
C26—N2—C25	108.56 (12)	C15—C16—H16	120.6
C26—N2—C27	108.21 (12)	C16—C17—C12	120.75 (13)
C25—N2—C27	108.21 (11)	C16—C17—H17	119.6
C2—C1—C10	120.30 (11)	C12—C17—H17	119.6
C2—C1—C11	119.87 (11)	C19—C18—C23	118.91 (13)
C10—C1—C11	119.62 (10)	C19—C18—N1	122.47 (11)
O1—C2—C1	118.17 (11)	C23—C18—N1	118.32 (11)
O1—C2—C3	121.60 (11)	C20—C19—C18	120.01 (12)
C1—C2—C3	120.22 (12)	C20—C19—H19	120.0
C4—C3—C2	120.00 (12)	C18—C19—H19	120.0
C4—C3—H3	120.0	C19—C20—C21	121.01 (13)
C2—C3—H3	120.0	C19—C20—H20	119.5
C3—C4—C5	121.48 (12)	C21—C20—H20	119.5
C3—C4—H4	119.3	C22—C21—C20	119.17 (14)
C5—C4—H4	119.3	C22—C21—H21	120.4
C4—C5—C6	122.23 (12)	C20—C21—H21	120.4
C4—C5—C10	118.96 (12)	C21—C22—C23	120.57 (13)
C6—C5—C10	118.79 (12)	C21—C22—H22	119.7
C7—C6—C5	121.37 (12)	C23—C22—H22	119.7
C7—C6—H6	119.3	C22—C23—C18	120.31 (13)
C5—C6—H6	119.3	C22—C23—H23	119.8
C6—C7—C8	119.72 (12)	C18—C23—H23	119.8
C6—C7—H7	120.1	O2—C24—H24A	109.5
C8—C7—H7	120.1	O2—C24—H24B	109.5
O2—C8—C9	124.82 (12)	H24A—C24—H24B	109.5
O2—C8—C7	114.39 (11)	O2—C24—H24C	109.5
C9—C8—C7	120.79 (12)	H24A—C24—H24C	109.5
C8—C9—C10	120.14 (12)	H24B—C24—H24C	109.5
C8—C9—H9	119.9	N2—C25—C26^i^	110.77 (12)
C10—C9—H9	119.9	N2—C25—H25A	109.5
C5—C10—C9	119.01 (11)	C26^i^—C25—H25A	109.5
C5—C10—C1	118.81 (11)	N2—C25—H25B	109.5
C9—C10—C1	122.18 (11)	C26^i^—C25—H25B	109.5
N1—C11—C12	117.33 (11)	H25A—C25—H25B	108.1
N1—C11—C1	126.31 (12)	N2—C26—C25^i^	110.40 (11)
C12—C11—C1	116.14 (11)	N2—C26—H26A	109.6
C13—C12—C17	118.81 (12)	C25^i^—C26—H26A	109.6
C13—C12—C11	120.47 (11)	N2—C26—H26B	109.6
C17—C12—C11	120.62 (12)	C25^i^—C26—H26B	109.6
C14—C13—C12	121.12 (13)	H26A—C26—H26B	108.1
C14—C13—H13	119.4	N2—C27—C27^i^	110.45 (7)
C12—C13—H13	119.4	N2—C27—H27A	109.6
C15—C14—C13	118.53 (13)	C27^i^—C27—H27A	109.6
C15—C14—H14	120.7	N2—C27—H27B	109.6
C13—C14—H14	120.7	C27^i^—C27—H27B	109.6
C14—C15—C16	121.86 (13)	H27A—C27—H27B	108.1
C14—C15—Cl1	118.57 (12)		
			
C10—C1—C2—O1	−179.95 (12)	C2—C1—C11—C12	81.97 (15)
C11—C1—C2—O1	5.43 (19)	C10—C1—C11—C12	−92.69 (13)
C10—C1—C2—C3	−0.9 (2)	N1—C11—C12—C13	−147.08 (12)
C11—C1—C2—C3	−175.50 (12)	C1—C11—C12—C13	27.86 (16)
O1—C2—C3—C4	−177.23 (13)	N1—C11—C12—C17	29.38 (16)
C1—C2—C3—C4	3.7 (2)	C1—C11—C12—C17	−155.68 (11)
C2—C3—C4—C5	−2.2 (2)	C17—C12—C13—C14	−2.25 (18)
C3—C4—C5—C6	179.21 (14)	C11—C12—C13—C14	174.28 (11)
C3—C4—C5—C10	−2.1 (2)	C12—C13—C14—C15	1.67 (19)
C4—C5—C6—C7	175.43 (14)	C13—C14—C15—C16	0.5 (2)
C10—C5—C6—C7	−3.2 (2)	C13—C14—C15—Cl1	−179.35 (10)
C5—C6—C7—C8	−0.4 (2)	C14—C15—C16—C17	−2.0 (2)
C24—O2—C8—C9	−5.2 (2)	Cl1—C15—C16—C17	177.82 (10)
C24—O2—C8—C7	174.25 (14)	C15—C16—C17—C12	1.4 (2)
C6—C7—C8—O2	−175.83 (13)	C13—C12—C17—C16	0.67 (18)
C6—C7—C8—C9	3.7 (2)	C11—C12—C17—C16	−175.85 (11)
O2—C8—C9—C10	176.23 (12)	C11—N1—C18—C19	59.63 (17)
C7—C8—C9—C10	−3.2 (2)	C11—N1—C18—C23	−126.69 (13)
C4—C5—C10—C9	−175.10 (12)	C23—C18—C19—C20	−1.1 (2)
C6—C5—C10—C9	3.62 (18)	N1—C18—C19—C20	172.57 (12)
C4—C5—C10—C1	4.86 (18)	C18—C19—C20—C21	1.4 (2)
C6—C5—C10—C1	−176.42 (12)	C19—C20—C21—C22	−0.4 (2)
C8—C9—C10—C5	−0.45 (19)	C20—C21—C22—C23	−0.9 (2)
C8—C9—C10—C1	179.60 (12)	C21—C22—C23—C18	1.2 (2)
C2—C1—C10—C5	−3.40 (18)	C19—C18—C23—C22	−0.2 (2)
C11—C1—C10—C5	171.24 (11)	N1—C18—C23—C22	−174.12 (12)
C2—C1—C10—C9	176.56 (12)	C26—N2—C25—C26^i^	−56.40 (14)
C11—C1—C10—C9	−8.81 (18)	C27—N2—C25—C26^i^	60.82 (16)
C18—N1—C11—C12	179.94 (11)	C25—N2—C26—C25^i^	60.43 (14)
C18—N1—C11—C1	5.59 (18)	C27—N2—C26—C25^i^	−56.79 (16)
C2—C1—C11—N1	−103.62 (15)	C26—N2—C27—C27^i^	61.24 (19)
C10—C1—C11—N1	81.72 (16)	C25—N2—C27—C27^i^	−56.21 (19)

Symmetry codes: (i) −*x*+1, *y*, −*z*+3/2.

### Hydrogen-bond geometry (Å, °)

**Table:**

*D*—H···*A*	*D*—H	H···*A*	*D*···*A*	*D*—H···*A*
O1—H1···N2^ii^	0.89 (2)	1.86 (2)	2.7401 (18)	167.2 (18)
C20—H20···Cl1^iii^	0.95	2.78	3.6071 (17)	146

Symmetry codes: (ii) −*x*+1, −*y*, −*z*+1; (iii) *x*+1/2, −*y*+1/2, *z*+1/2.
